# Meralgia Paresthetica after Prone Positioning Ventilation in the Intensive Care Unit

**DOI:** 10.1155/2016/7263201

**Published:** 2016-09-26

**Authors:** Christian Svendsen Juhl, Martin Ballegaard, Morten H. Bestle, Peer Tfelt-Hansen

**Affiliations:** ^1^Department of Anesthesiology, Bispebjerg Hospital, University of Copenhagen, Copenhagen, Denmark; ^2^Department of Clinical Neurophysiology, Rigshospitalet, University of Copenhagen, Copenhagen, Denmark; ^3^Department of Anesthesiology, Intensive Care Unit, Nordsjaellands Hospital, University of Copenhagen, Hillerød, Denmark; ^4^Department of Neurology, Nordsjaellands Hospital, University of Copenhagen, Hillerød, Denmark; ^5^Department of Neurology, Zealand University Hospital in Roskilde, Roskilde, Denmark

## Abstract

Meralgia paresthetica (MP) is a mononeuropathy of the lateral femoral cutaneous nerve (LFCN) caused by external compression of the nerve during its course close to the anterior superior iliac spine. We present a case of a patient with acute respiratory distress induced by* Legionella* pneumonia who was admitted to the intensive care unit (ICU) for mechanical ventilation. In the ICU, the patient received one session of prone position ventilation for 8.5 consecutive hours. At evaluation six months later, the patient reported persistent bilateral numbness of the anterolateral thigh, which he complained had begun right after he woke up at the ICU. He was referred for further neurological and neurophysiological examination and was diagnosed with bilateral MP, a condition never previously described as a complication to mechanical ventilation in prone position in the ICU.

## 1. Introduction

Meralgia paresthetica (MP), or Bernhardt-Roth syndrome, is a mononeuropathy of the lateral femoral cutaneous nerve (LFCN), resulting in hyposensitivity of the anterolateral thigh and sometimes neuropathic pain [1]. Since the LFCN is a sensory nerve, no motor symptoms are seen in MP. The condition is caused by compression of the LFCN, either idiopathic or iatrogenic. The idiopathic form is typically associated with diabetes mellitus, advancing age, and obesity [2]. The iatrogenic form is, among others, seen as a complication to surgery with the patient placed in prone position but has, to our knowledge, never been described as a complication to mechanical ventilation in prone position in the intensive care unit (ICU) [3]. The mechanism of the iatrogenic MP is direct external compression of the LFCN.

Prone positioning ventilation is frequently used in intensive care to improve blood oxygenation in patients with severe pneumonia and/or acute respiratory distress syndrome (ARDS) [4]. We present a case of a patient who developed bilateral meralgia paresthetica after 8.5 hours of prone positioning ventilation in the ICU.

## 2. Case Presentation

A 57-year-old male, with no prior medical history, was admitted to a hospital for cough, fever, and respiratory distress. One day previously he had returned from a two-week holiday in Thailand. He had a body mass index (BMI) of 23 kg/m^2^, smoked 12 to 20 cigarettes per day, and denied alcohol abuse.

Chest radiograph revealed infiltrates in the left lower lobe and in the right hilar region. Antibiotic treatment was initiated with ceftriaxone and ciprofloxacin. As urine analysis came out positive for* Legionella* pneumoniae antigen, clarithromycin was added to the antibiotic treatment. Oxygen saturation deteriorated despite oxygen treatment with a high-flow oxygen device, and he was admitted to ICU for mechanically assisted ventilation. On day 2, it was still not possible to reduce inspiratory oxygen fraction (F_i_O_2_) to less than 0.95 without compromising oxygen saturation, and prone position ventilation was started and continued for 8.5 hours in an attempt to improve the pulmonary ventilation/perfusion profile.

**Figure 1:**
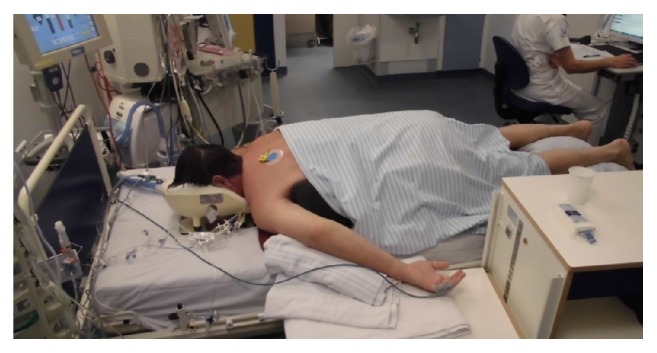
Mechanical ventilation of the patient in prone position.

During prone position ventilation, the patient was positioned with the forehead and chin resting on a head rest (Figure 1). Two supporting pads were placed under the upper thorax and the hips, respectively, leaving the abdomen hanging free, without pressure on the penis or scrotum. A third supporting pad was placed under the lower legs. Nursing care included alternately hourly bending and extension of shoulders, elbows, hips, and knees, slight turning of the head, and observation for skin pressure marks.

During the following four days, F_i_O_2_ was successfully reduced to 0.35 without further need for prone positioning ventilation. Extubation was performed one week after admission, and on day 10 he was discharged from the ICU.

Six months after hospital discharge, a lung spirometry was performed. The spirometry was normal, but the patient complained of numbness on the lateral side of both thighs, which he had noticed already when he woke up in the intensive care unit six months earlier. He was referred for further neurological and neurophysiological examination as bilateral meralgia paraesthetica was suspected.

Eleven months after the initial admission, the diagnosis was confirmed with a sensory nerve conduction study of the LFCN, recording from the nerve at the inguen using near-nerve needles and using surface cutaneous stimulation at multiple transverse positions on the thigh. Sensory nerve action potentials were found at a few positions on both sides, only, documenting a severe axonal loss in both nerves. Electromyography from the quadriceps muscle was without neurogenic changes, suggesting no involvement of the lumbar plexus. Further motor nerve conduction studies of tibial and peroneal nerves and sensory nerve conduction studies of both sural nerves were without signs of polyneuropathy, and a median and tibial somatosensory evoked potential study was without signs of central or peripheral conduction abnormalities. Magnetic resonance imaging (MRI) of the thoracolumbar spine was conducted but showed no lumbar prolapse and no lesions of the thoracic part of the spinal cord or the cauda equina.

## 3. Discussion

The patient in the present case developed MP after a single session of prone positioning ventilation lasting 8.5 hours. The case illustrates an unusually severe course of the condition, as no spontaneous improvement currently has occurred after more than ten months.

LFCN originates from the second and third lumbar spinal nerves traversing the lumbar plexus before it passes along the posterolateral part of the major psoas muscle and crosses the ilium towards the anterior superior iliac spine (ASIS). Anatomical variations exist in the course of the nerve and its relation to ASIS. Aszmann et al. found that 54% of dissected human specimens had an anatomical variation that was susceptible to mechanical trauma [5]. MP is a well-known complication to prone positioning during lumbar spine surgery [3, 6]. Three different studies involving 110, 105, and 252 patients found an incidence of MP following spine surgery in 12%, 20%, and 24% of the patients, respectively [6–8]. Risk factors include surgery longer than 3.5 hours and degenerative spinal disorders related to the second and third spinal nerve root [3]. The role of BMI as a prognostic factor for developing MP after prone position surgery is uncertain. Yang et al. found higher incidence of MP and a longer recovery period among patients with a greater BMI [8]. On the other hand, Gupta et al. found higher incidence of MP in thinner patients and attribute this to higher sensitivity to direct compression of the LFCN [6]. The patient in the present study developed MP despite normal BMI of 23 kg/m^2^.

Iatrogenic MP after surgery is usually self-limiting on conservative treatment, and in most cases complete recovery is seen within weeks or months [6, 8]. However, in cases with severe pain that persists despite nonoperative treatment, surgical treatment may be considered [9, 10]. The patient in the present case had persistent symptoms even after six months. This could be due to more severe nerve damage caused by prone positioning for a relatively long duration (8.5 hours) compared to the duration of the prone positioning during spinal surgery. In a recent meta-analysis of the efficacy of prone positioning in adults with ARDS, the authors concluded that mortality rates were reduced only when the daily duration of prone positioning was >12 hours [4]. As a result, the duration of the prone position is critical when used to treat ARDS, and it can be anticipated that, in the future, prone position ventilation will be used more frequently and with longer duration in the ICU.

MP has, to our knowledge, never been described as a complication to prone positioning ventilation, even though prone positioning has been used in critical care for decades. MP is probably an underdiagnosed condition in patients who are mechanically ventilated in prone position in the ICU. The condition is usually a clinical diagnosis and is rarely confused with lumbar root lesions with the cutaneous distribution of the L3 nerve root spirals from the lateral thigh across the anterior part to the medial part, whereas the sensory distribution of LFCN is distributed vertically on the lateral side of the thigh.

Most patients make full recovery without any specific treatment. However, with increasing use and longer duration of prone position ventilation in the ICU, the risk of developing MP should be considered. We suggest that special care should be taken to avoid unnecessary compression of the ASIS when a sedated patient is placed in prone position for mechanical ventilation in the ICU and that special soft padding be used for supporting the hip.

## 4. Conclusion

MP is a mononeuropathy of the LFCN. The iatrogenic form is caused by direct compression of the nerve, and anesthetized patients are at risk when placed in prone position for several hours. MP has not previously been described as a complication to prone positioning ventilation in the ICU and is probably underdiagnosed. This case serves as a reminder to avoid unnecessary pressure to the ASIS when placing a patient with ARDS in prone position for mechanical ventilation, as severe cases of MP do not respond sufficiently to conservative treatment.
